# Abstract of the Proceedings of the Ninth Annual Session of the American Medical Association, Held in Detroit, May 6, 7, 8 and 9, 1856

**Published:** 1856-06

**Authors:** 


From the Medical Counselor.

*Abstract of the Proceedings of the Ninth Annual Session of the American Medical Association, held in Detroit, May* 6, 7, 8 *and* 9, 1856.

*Prepared by the Editor.*

The Association met at 11 o'clock, A. M., on the 6th, the President, Dr. G. B. Wood, of Pa., presiding.

Dr. Pitcher, of Michigan, in behalf of the Committee of Arrangements, welcomed the Association to Detroit, in a few very pertinent remarks.

Dr. Wistar, of Pa., announced the names and residence of the delegates in attendance, amounting to about 200, afterwards increased to a total number of 300.

The following is a list of the Ohio Delegation:

J. W. Sayles, Cleveland; B. Sheldon, do; W. H. Philips, Kenton; John C. Maggin, Fayetteville; Benj. Tappan, Steubenville; Lewis Slusser, Canal Fulton; Benj. Stanton, Salem; S. G. Armor, Cincinnati; Richard Gundry, Columbus; C. H. Swayne, Toledo; F. F. Hurxthal, Massillon; H. J. Dunahoo, Sandusky; D. Tilden, do; A. Austin, do; Geo. Mendenhall, Cincinnati; W. L. Schenk, Franklin; G. Volney Dorsey, Piqua; R. R. McMeens, Sandusky; S. Bonner, Cincinnati; Adam Mosgrove, Urbana; John A. Smith, Piqua; G. F. Mitchell, Mansfield; B. Neff, Piqua; J. N. Gard, Greenville; A. W. Munson, Kenton; R. D. Mussey, Cincinnati; L. C. Rives, do; Wm. Clendenin, do; Henry Spillman, Medina; H. O. Mack, Shelby; Charles Cochran, Sandusky; T. W. Gordon, Georgetown; E. H. Sibley, Medina; Alfred Ayres, Greenville; L. D. Griswold, Elyria; Wm. Trevitt, Columbus; Chas. Robertson, McConnelsville; J. P. Henderson, Newville; Isaac Heustis, Chester Hill; Smith Branson, do; R. Hills, Columbus; Abel Carey, Salem; C. McDermont, Dayton; H. Gatch Cary, do; J. W. White, Stockfort, Morgan Co.; W. W. Jones, Toledo.

On motion of Dr. Thomson, of Delaware, a recess of fifteen minutes was taken to allow the delegates from the respective States to report one member from each State represented, as a committee to nominate officers for the ensuing year.

At the expiration of the recess, the Association was called to order, and the different State delegations then reported their choice, respectively, of delegate to serve on the nominating committee, which was constituted as follows:

Maine, N. P. Monroe; New Hampshire, H. Peirce; Vermont, C. L. Allen; Massachusetts, H. H. Childs; Rhode Island, J. E. Warren; Connecticut, David Harrison; New York, William Rockwell; New Jersey, L. A. Smith; Pennsylvania, John Neill; Delaware, J. W. Thomson; Maryland, P. Wroth; South Carolina, E. Geddings; Tennessee, J. B. Lindsley; Kentucky, W. S. Sutton; Minnesota, C. W. LeBoutillier; Michigan, M. Gunn; Ohio, Thos. W. Gordon; Indiana, Dr. Winton; Illinois, H. Noble; Wisconsin, W. H. Brisbane.

Dr. Pitcher, from the committee of Arrangements announced the hours of session proposed by the committee to be from 9 A. M., to 12.30 P. M., and from 2 P. M. to 5 P. M. These hours were adopted.

The President announced the death of the eminent Dr. John C. Warren, of Boston, Mass., in that city, on Sunday morning.

Dr. Childs, of Mass., felt compelled to say a few words in this connection. He had been associated with the deceased for more than half a century, and should feel that he had been derelict of duty if he neglected to speak in his laudation. Dr. Warren was the nephew of Joseph Warren, who fell gloriously at the battle of Bunker Hill. He was at the head of his profession in Massachusetts—had been President of the State Medical Society, and occupant of other elevated medical positions. His professional reputation was high, and his personal reputation spotless. His fame was not confined to Massachusetts. Though devoted to medical science, he was not limited to that alone, but paid attention to every branch of literature and art. If young members of the profession would be useful and eminent, they should follow the example of Dr. J. C. Warren. To the older, the speaker would point out Dr. W.'s moral character as an exemplar. Such a life as his inevitably terminates in a death beautified by a surety of eternal happiness.

Dr. Gross, of Kentucky, made some remarks eulogistic of the deceased. He alluded to his high reputation—a reputation, he observed, not confined to America, but extending to every corner of the civilized world. Dr. Warren was the Nestor of American surgery. Dr. G. concluded by offering the following:

*Resolved*, That a committee of five be appointed to draft resolutions expressive of the feelings of this Association at the loss of their late associate, Dr. John C. Warren.

The resolution was adopted, and the President appointed as such committee, Dr. Gross of Kentucky, Dr. Childs of Massachusetts, Dr. Wood of New York, Dr. Pitcher of Michigan, and Dr. Geddings of South Carolina.

On motion, the Association adjourned to 2 P. M.

### AFTERNOON SESSION.

The President called the Association to order at 2 o'clock.

The committee on Nominations submitted the following report:

The committee on Nominations unanimously nominate the following officers of the American Medical Association for the ensuing year:

*President*—Dr. Zina Pitcher, of Detroit.

*Vice-Presidents*—Drs. Thomas W. Blatchford, of New York; Wm. K. Bowling, of Tennessee; E. Geddings, of South Carolina; W. H. Brisbane, of Wisconsin.

*Secretaries*—Drs. Wm. Brodie, of Michigan; R. C. Foster, of Tennessee.

*Treasurer*—Dr. Casper Wister, of Pennsylvania.

The report was accepted, and the nominations unanimously confirmed.

On motion of Dr. Atlee, of Pennsylvania, the President was requested to deliver his annual address.

The retiring President then delivered his Valedictory, which was replete with fervid eloquence and sound advice, and instruction, in regard to the honor and usefulness of the profession. One thousand extra copies were afterwards ordered to be printed. As pertinent to a subject of considerable interest to the profession in Ohio at the present time, we will publish so much of the address as relates to the subject of Medical Ethics. [Will be inserted in the next number.]

At the conclusion of the address, on motion of Dr. Atlee, of Pennsylvania,

*Resolved*, That the thanks of the Association be presented to our late President for the able and interesting parting address he has just delivered, and that he be requested to present to the Committee of Publication, a copy for preservation in our transactions.

On motion of Dr. Atlee, of Pa.,

*Resolved*, That a committee of three be appointed to inform the President and Vice Presidents elect of their election, and conduct them to their seats.

The President appointed, as such committee, Drs. Atlee, of Pa., Rives, of Ohio, and Sutton, of Ky.

Upon taking the chair, Dr. Pitcher said:

"Although fully aware of my indebtedness, for this distinction, to your observance of a custom equivalent in force to positive law, of selecting your presiding officer in each successive year, from the State in which the meeting of the Association is held, I feel myself more honored by your partiality, than if I had received the same mark of respect from any other body of men known to the annals of our country.

"This sentiment of regard for the body towards which I now hold, by this act of yours, so delicate and interesting a relation, has been inspired by a contemplation of the ideal of the physician, and strengthened by my growing acquaintance with the individuals which compose it.

"Being unaccustomed to preside in deliberative assemblies, I shall throw myself upon the indulgence of the Association, and rely upon the kindness and intelligent coöperation of the individual members for assistance, in performing the duties of the chair.

"Whilst thanking you most cordially for this expression of confidence, I can only assure you that such abilities as I possess shall be devoted to the prosperity of the Association and the harmony of its proceedings."

On motion of Dr. Gunn, of Mich.,

*Resolved,* That the resolution passed at St. Louis, requiring a majority of the committee on Publication to be appointed from residents of the place where the meeting is held, be repealed.

At the request of Dr. Gross, of Ky., his report upon "The Causes that Retard Medical Education and Literature," was made the special order for Wednesday, at 10 o'clock.

Dr. Palmer, of Ill., from the committee on Prize Essays and Volunteer Communications, submitted the following:

"The committee on Prize Essays and Volunteer Communications" report, that some months since they issued a card, which was extensively published in the medical journals, setting forth the terms upon which essays intended for prizes would be received; but that the number of papers has been but four.

By referring to the past records of the Association, it is found that the numbers received by preceeding committees have been in 1852, sixteen; in 1853, fifteen; in 1854, nine; in 1855, six; in 1856, four. Your committee beg leave to call attention to this almost regular and quite rapid decrease in the number of essays presented, for the purpose of having the Association consider whether there be not danger that the number which may hereafter be furnished will be so small as to afford insufficient range of comparison and choice to cause the preference shown to be much valued, if, indeed, presentations do not cease altogether, and whether any means should be devised for preventing such result.

The essays received by your committee have been subjected to a careful examination; and while admitting that they all possess a degree of merit which would render them suggestive and useful, if given so the profession, still, in their opinion, but one manifests that evidence of careful and laborious investigation, that wide scope and rigid accuracy of logical reasoning, that chasteness of expression and artistic skill in the presentation of the subject, as to furnish sufficient claim for awarding a prize by this body.

But one prize is therefore awarded. The essay selected for this honor bears the title:—"An Essay on the Arterial Circulation."

It is regarded by the committee as possessing the merits just alluded to, and while not wishing to give an unqualified indorsement of all the views which it contains, they regard it as possessing not only interest in its physiological and scientific relations, but also real value in its pathological and practical bearings.

The production has considerable length, and by the fullness with which the views advanced are discussed, it partakes as much of the nature of a treatise, as an essay. It has, at least, one quality which Lord Bacon considered necessary to a treatise, as distinguished from an essay,—it required a degree of leisure on the part of the writer, and will require the same on the part of the reader for him fully to appreciate its value.

The essay bears the motto—"*Una est veritas.*"

(Signed) A. B. PALMER, Ch'n.
SAMUEL DENTON,
SILAS H. DOUGLASS,
AB'M SAGER,
E. ANDREWS.

On breaking the seal of the accompanying packet, Dr. Henry Hartshorn, of Philadelphia, Pa., was found to be the successful essayist.

The report was accepted.

Dr. Blatchford, of N. Y., from the committee on "Hydrophobia, and the Connection of the Season of the year with its Prevalence," read a report thereon. The committee, in conclusion, submitted the following resolution, which was adopted:

*Resolved*, That the Secretary transmit to the Governor of each State a copy of the statistical part of this report, with the respectful request that he would bring the subject before the Legislature of the State over which he presides, that in their wisdom they may devise and unite upon a plan by which the evil may be mitigated, if not removed.

The committee on Nominations reported in favor of holding the next annual meeting of the Association at Nashville, Tenn.

Dr. Gross, of Ky., moved to strike out "Nashville, Tenn.," and insert "Louisville, Ky." He thought Nashville at present difficult of access.

Dr. Geddings, of S. C., and Lindsley, of Tenn., advocated the adoption of the report.

Dr. Gross withdrew his amendment and the report was adopted.

[TO BE CONTINUED.]

